# Past, current, and potential treatments for cryptosporidiosis in humans and farm animals: A comprehensive review

**DOI:** 10.3389/fcimb.2023.1115522

**Published:** 2023-01-24

**Authors:** Shahbaz M. Khan, William H. Witola

**Affiliations:** Department of Pathobiology, College of Veterinary Medicine, University of Illinois Urbana-Champaign, Urbana, IL, United States

**Keywords:** *Cryptosporidium*, cryptosporidiosis, treatment, prevention, drug discovery, protozoa, diarrhea

## Abstract

The intracellular protozoan parasite of the genus *Cryptosporidium* is among the leading causes of waterborne diarrheal disease outbreaks throughout the world. The parasite is transmitted by ingestion of infective oocysts that are highly stable in the environment and resistant to almost all conventional disinfection methods and water treatments. Control of the parasite infection is exceedingly difficult due to the excretion of large numbers of oocysts in the feces of infected individuals that contaminate the environment and serve as a source of infection for susceptible hosts including humans and animals. Drug development against the parasite is challenging owing to its limited genetic tractability, absence of conventional drug targets, unique intracellular location within the host, and the paucity of robust cell culture platforms for continuous parasite propagation. Despite the high prevalence of the parasite, the only US Food and Drug Administration (FDA)-approved treatment of *Cryptosporidium* infections is nitazoxanide, which has shown moderate efficacy in immunocompetent patients. More importantly, no effective therapeutic drugs are available for treating severe, potentially life-threatening cryptosporidiosis in immunodeficient patients, young children, and neonatal livestock. Thus, safe, inexpensive, and efficacious drugs are urgently required to reduce the ever-increasing global cryptosporidiosis burden especially in low-resource countries. Several compounds have been tested for both *in vitro* and *in vivo* efficacy against the disease. However, to date, only a few experimental compounds have been subjected to clinical trials in natural hosts, and among those none have proven efficacious. This review provides an overview of the past and present anti-*Cryptosporidium* pharmacotherapy in humans and agricultural animals. Herein, we also highlight the progress made in the field over the last few years and discuss the different strategies employed for discovery and development of effective prospective treatments for cryptosporidiosis.

## 1 Introduction

### 1.1 History

The intracellular protozoan parasite *Cryptosporidium* is one of the most common parasitic pathogens causing enteric disease in humans and in a broad range of animals worldwide (Chalmers, 2014). First recognized and described briefly in 1907 by Ernest Tyzzer in the gastric glands of the common mouse (Tyzzer, 1907), *Cryptosporidium* was later described in greater detail in 1910, again from histological preparations from the murine gastric mucosa (Tyzzer, 1910). Tyzzer proposed the name *Cryptosporidium muris* for the parasite (Tyzzer, 1907; Tyzzer, 1910). In 1912, Tyzzer described another species with smaller oocysts than those of *C. muris* in the small intestine of experimentally infected laboratory mice, which he named *Cryptosporidium parvum* (Tyzzer, 1912). Although *Cryptosporidium* was subsequently identified in a wide range of domesticated animals, this genus of parasites only gained importance in the 1970s (after almost 7 decades from its initial discovery), when the parasite was found to be linked to gastrointestinal disease in humans and farm animals (Panciera et al., 1971; Meuten et al., 1974; Meisel et al., 1976; Nime et al., 1976). In the 1980s, cryptosporidiosis gained more widespread recognition after reports of fatal cryptosporidiosis in AIDS patients (Soave et al., 1984), zoonotic cryptosporidiosis in immunocompetent and immunodeficient humans (Current et al., 1983), waterborne human diarrheal outbreaks (D'Antonio et al., 1985; Hayes et al., 1989), and diarrheal disease in children (Sallon et al., 1988) and animals (Tzipori et al., 1980; Moon and Bemrick, 1981; Angus et al., 1982). In 1993, *Cryptosporidium* caused the largest documented drinking water outbreak in US history, which affected an estimated 403,000 people in Milwaukee, Wisconsin, and resulted in over $96 million in combined healthcare costs and productivity losses (Mac Kenzie et al., 1994; Hoxie et al., 1997; Corso et al., 2003). The enormity of the Milwaukee outbreak sparked concern among the public and attracted generous funds for *Cryptosporidium* research from governmental agencies all over the world during the next decade. This resulted in further advances in our knowledge about the basic biology of the parasite and the development of reliable molecular detection tools for estimating the global burden of the disease.

### 1.2 Life cycle

**Figure 1:**
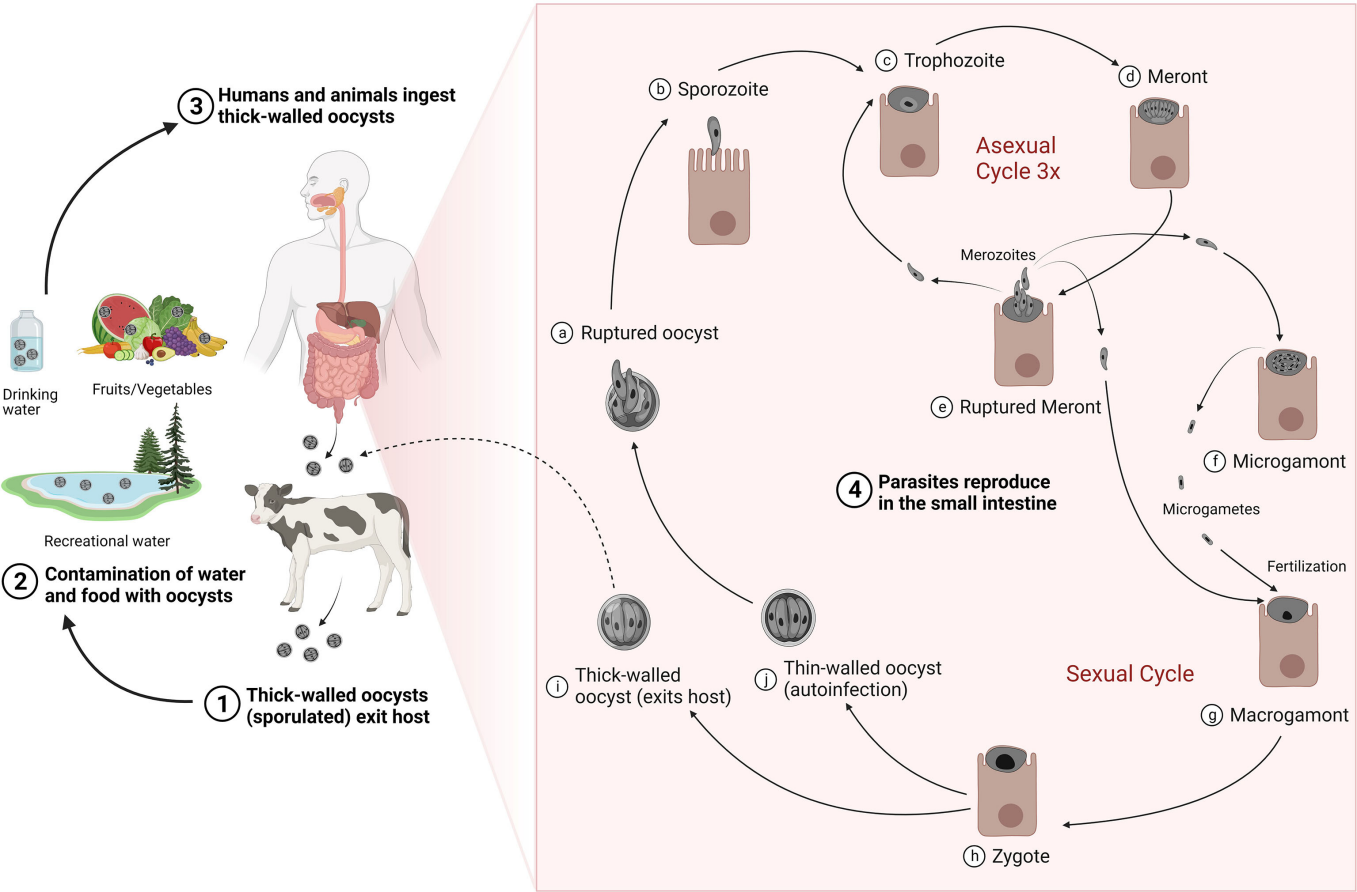
Life cycle and transmission of *Cryptosporidium*. Thick-walled sporulated oocysts are released in the feces of infected hosts (1) that contaminate food and water sources (2). Transmission occurs mainly by ingestion of contaminated water or food by susceptible hosts (3). Following ingestion, oocyst ruptures (4a) to release four sporozoites (4b). Sporozoites exhibit gliding motility, enter the host epithelial cells and mature into trophozoites (4c), which undergo three rounds of asexual multiplication to produce meronts (4d) that invariably release eight merozoites (4e). Merozoites released from the third round of asexual proliferation give rise to the sexual stages upon reinvasion of host cells: the male microgamonts (4f) and the female macrogamonts (4g). Microgametes released from the microgamont penetrate and fertilize macrogamonts to form diploid zygotes (4h). The zygotes undergo meiosis and sporogony generating either thin-walled (4i) or thick-walled (4j) oocysts, each containing four haploid sporozoites. Thick-walled oocysts are released into the lumen of the intestine and excreted into the environment, where they are instantly infectious. The thin-walled oocysts, in contrast, excyst to cause autoinfection in the same host. Adapted with modification from (CDC, 2019). Created with BioRender.com.

The life cycle of *Cryptosporidium* is direct and complex (**Figure 1**), consisting of both asexual multiplication and sexual reproduction phases within a single host that culminate in the production of environmentally resistant oocysts (Current and Garcia, 1991). Following ingestion of sporulated thick-walled oocysts, four infectious sporozoites are released from each oocyst that attach to the apical surface of intestinal epithelial cells, and then actively invade the host cell membrane to form an intracellular but extracytoplasmic parasitophorous vacuole (Current and Reese, 1986). Within the vacuole, sporozoites mature into trophozoites, which undergo three rounds of asexual proliferation, followed by a single generation of sexual stages to generate either thin-walled or thick-walled oocysts, each containing four haploid sporozoites (Current and Reese, 1986; English et al., 2022). Thick-walled oocysts containing two-layered membranes are environmentally resistant and are passed out of the body in feces, where they are immediately infectious for other susceptible hosts. Thin-walled oocysts rupture in the intestinal lumen, releasing naked infectious sporozoites that autoinfect other enteric cells to ensure continued infection of the same host.

### 1.3 Transmission

In general, cryptosporidiosis is transmitted through the fecal-oral route (**Figure 1**) and contact with animals, manure or contaminated food and water is believed to lead to infections in humans (Xiao et al., 2004). Transmission in animals mainly occurs *via* ingestion of oocysts excreted by infected animals especially neonates in overcrowded or mixed housing facilities. Manure produced by livestock, especially cattle, is an important source of infection to both animals and people and it has been estimated that the global *Cryptosporidium* load in livestock manure is approximately 3.2 × 10^23^ oocysts per year (Vermeulen et al., 2017). Oocysts are highly stable in the environment and resistant to almost all conventional disinfection methods and water treatments such as chlorination (Fayer et al., 2000). Indeed, these persistent parasites have been found to be responsible for majority of the global protozoal water outbreaks that occurred from 2004–2010 (Karanis, 2018) and pose the biggest pathogen threat to the water industry (Chalmers, 2012). In the United States, exposure to treated recreational water such as swimming pools and water playgrounds was responsible for nearly 35% of the reported cryptosporidiosis outbreaks resulting in almost 57% cases during 2009–2017 (Gharpure et al., 2019). In addition to treated recreational water, contact with infected cattle (~15%), and contact with infected persons in childcare settings (~13%) were the other predominant causes of these outbreaks (Gharpure et al., 2019). *Cryptosporidium* is also recognized as an important foodborne pathogen, being responsible for more than 40 documented foodborne outbreaks to date, and more than 8 million cases of foodborne illnesses annually (Zahedi and Ryan, 2020). However, these numbers may be highly under-reported due to the lack of proper surveillance and the difficulties in tracing the source of foodborne disease outbreaks. Food can be contaminated at any point along the food production chain (during processing, distribution, or preparation) by direct contact with infected food handlers or by indirect exposure to water, preparation surfaces, equipment, or utensils contaminated with oocysts. Raw unpasteurized milk, unpasteurized apple cider, and salads are most associated with foodborne outbreaks of cryptosporidiosis (Gharpure et al., 2019; Zahedi and Ryan, 2020).

## 2 Cryptosporidiosis global disease burden in humans and animals

*Cryptosporidium* spp. enjoy high parasitic success due to their wide host range, low infective threshold, high excretion of resistant oocysts from infected individuals, and water-borne route of transmission (Innes et al., 2020). Protozoa of this genus are associated with diarrheal disease throughout the world with a higher incidence in developing countries (Shirley et al., 2012). Cryptosporidiosis is a major cause of public health concern in developed countries as well, with reported cases on the rise mainly due to the leading role of *Cryptosporidium* in causing waterborne outbreaks (Gharpure et al., 2019). Unfortunately, the global burden of cryptosporidiosis is likely to be underestimated, due to the lack of cheap and consistent methods of diagnosis, under-recognized disease in immunocompetent patients, lack of proper surveillance in developed countries, and difficulties observed in measuring the impact of the disease in poor-resource areas. Many infected individuals either do not exhibit symptoms or exhibit mild symptoms due to a self-limiting illness, and such infections often go unrecognized. There is a wide range of disease severity that is affected by the host’s age, nutritional, and immune status, and perhaps by the parasite species and subtype (Shirley et al., 2012). Differences in sensitivity of methods, type of diagnostics used, and study populations have resulted in a large variation in burden estimates for diarrhea from *Cryptosporidium* infection in humans and animals. However, the recent advances in knowledge and the development of highly sensitive and superior diagnostic and typing tools have improved our understanding of the epidemiology and true burden of the disease.

### 2.1 Humans

Out of the currently documented 44 species of *Cryptosporidium*, *Cryptosporidium hominis* (*C. hominis*) and *Cryptosporidium parvum* (*C. parvum*) are responsible for most human infections (Ryan et al., 2021). While *C. hominis* is primarily anthroponotic and only infects humans, *C. parvum* is a zoonotic parasite that can be transmitted between humans and animals. Apart from *C. hominis* and *C. parvum*, 21 other *Cryptosporidium* species and genotypes including *C. meleagridis*, *C. felis*, *C. canis*, *C. ubiquitum*, *C. cuniculus*, *C. ditrichi*, *C. erinacei*, *C. fayeri*, *C. scrofarum*, *C. tyzzeri*, *C. viatorum*, *C. muris*, *C. andersoni*, *C. suis*, *C. bovis*, *C. occultus*, *C. xiaoi*, horse genotype, chipmunk genotype I, skunk genotype, and mink genotype have also been reported in humans (Ryan et al., 2021).

Cryptosporidiosis is considered a high-risk and often lethal opportunistic disease for patients with compromised immune systems such as those suffering from HIV/AIDS (O'Connor R et al., 2011) or those receiving organ transplants (Danziger-Isakov, 2014; Bhadauria et al., 2015). The global prevalence of *Cryptosporidium* in HIV/AIDS patients was 10.09% during the period from 2007 to 2017 (Wang et al., 2018). However, the greatest burden of cryptosporidiosis occurs among young children living in less developed countries. *Cryptosporidium* prevalence is higher in such areas that lack proper sanitation facilities, mainly drinking water and sewage, which led the World Health Organization (WHO) to include it in the water sanitation and health program (WHO, 2009). Several epidemiological studies conducted in the previous decade have estimated the disease burden of diarrheal pathogens in developing countries. In the Global Enteric Multicenter Study (GEMS) conducted at seven sites in sub-Saharan Africa and South Asia, *Cryptosporidium* was found to be the main cause of linear growth faltering and the second leading cause of moderate-to-severe diarrhea in infants (0–11 months of age) (Kotloff et al., 2013; Nasrin et al., 2021). *Cryptosporidium* infection was also associated with a higher risk of mortality in diarrheic children aged 12–23 months who were admitted to hospitals (Kotloff et al., 2013). The 2016 Global Burden of Diseases, Injuries, and Risk Factors study (GBD) identified *Cryptosporidium* as a leading cause of diarrheal mortality in children younger than 5 years old with an estimated loss of 4.2 million disability-adjusted life years (DALYs) (Troeger et al., 2018). However, this study focused on acute illness alone, and as such, the number increased to 12.9 million DALYs, when long-term-effects of cryptosporidiosis such as growth retardation and cognitive defects were also considered (Khalil et al., 2018). Furthermore, the MAL-ED (Etiology, Risk Factors, and Interactions of Enteric Infections and Malnutrition and the Consequences for Child Health and Development Project) study carried out at eight sites in South America, sub-Saharan Africa, and Asia, found that *Cryptosporidium* along with four other pathogens exhibited the highest attributable burdens of diarrhea in community clinics in the first year of life (Platts-Mills et al., 2015).

### 2.2 Cattle

At least four main *Cryptosporidium* species infect cattle: *C. parvum*, *C. bovis*, *C. ryanae*, and *C. andersoni* (Lindsay et al., 2000; Santin et al., 2004; Fayer et al., 2005; Fayer et al., 2008), although other species have also been reported in sporadic cases, including *C. felis, C. hominis*, *C. suis, C. canis*, *C. scrofarum, C. tyzzeri, C. serpentis*, and *C*. *occultus* (formerly known as the *C. suis*-like genotype) (Robertson et al., 2014; Santin, 2020). The occurrence of *C. parvum*, *C. bovis*, *C. ryanae*, and *C. andersoni* in cattle follows an age-related pattern: the zoonotic *C. parvum* infects mostly pre-weaned calves, *C. bovis* and *C. ryanae* are found mostly in post-weaned calves, whereas *C. andersoni* is the predominant species found in heifers and adults (de Graaf et al., 1999; Santin et al., 2004; Fayer et al., 2006; Santin et al., 2008; Khan et al., 2010).

Cryptosporidiosis is one of the most important global causes of diarrhea in neonatal farm ruminants including calves. *Cryptosporidium* parasites invade intestinal epithelial cells and cause severe mucosal erosion resulting in villus shortening and fusion, and hypertrophy of crypts at small intestinal sites that lead to impaired digestion and increased intestinal permeability (Tzipori et al., 1983). The resulting diarrhea causes high production losses including mortality, reduced live weight gain, veterinary costs, and the added feeding and rearing costs for affected animals with slowed growth rates (Innes et al., 2020; Santin, 2020; Shaw et al., 2020). Cryptosporidiosis is recognized as endemic in cattle worldwide and the prevalence of bovine cryptosporidiosis varies substantially between countries, age groups, and studies, ranging from 11.7 to 78%, with the highest incidence reported in pre-weaned calves (Santin et al., 2004; Watanabe et al., 2005; Fayer et al., 2006; Maddox-Hyttel et al., 2006; Trotz-Williams et al., 2007; Khan et al., 2010; Amer et al., 2013; Thomson et al., 2017; Hatam-Nahavandi et al., 2019). An 18-month longitudinal study that focused on 2,545 dairy heifer calves from birth to weaning at 104 dairy operations in 13 US states identified at least 1 calf positive for *Cryptosporidium* at almost all operations (Urie et al., 2018b). Furthermore, the overall prevalence of *Cryptosporidium* in pre-weaned heifer calves was 43.1% with the disease more prevalent among young calves less than 2 weeks of age (63.3%) compared with calves older than 6 weeks (9.1%) (Urie et al., 2018a).

### 2.3 Small ruminants

Of the species infecting small ruminants, *C. parvum*, *C. ubiquitum*, *and C. xiaoi* are the most frequently detected species (Fayer and Santin, 2009; Fayer et al., 2010; Santin, 2020). In addition, *C*. *andersoni*, *C*. *bovis*, *C*. *ryanae*, *C*. *hominis*, *C*. *fayeri*, *C. baileyi*, and *C*. *suis* have been identified sporadically in sheep and goats (Hatam-Nahavandi et al., 2019; Santin, 2020).

*Cryptosporidium* causes significant morbidity and mortality in neonatal lambs and goat kids (de Graaf et al., 1999; Wright and Coop, 2007). Diarrhea resulting in reduced productivity and growth has been associated with *Cryptosporidium* infections in lambs and kids (Paraud and Chartier, 2012; Jacobson et al., 2016; Jacobson et al., 2018). *Cryptosporidium* shedding was also associated with less carcass weight and lowered dressing percentage in both symptomatic and apparently asymptomatic sheep on Australian farms (Jacobson et al., 2016). A wide range of *Cryptosporidium* prevalence based on microscopy and molecular detection methods has been reported in small ruminants worldwide ranging from 12.5% to 77.4% in lambs (Causape et al., 2002; Ryan et al., 2005; Castro-Hermida et al., 2007; Goma et al., 2007; Santin et al., 2007; Geurden et al., 2008) and from 4.8% to 70.8% in goat kids (Noordeen et al., 2001; Watanabe et al., 2005; Castro-Hermida et al., 2007; Goma et al., 2007; Geurden et al., 2008). As in cattle, *Cryptosporidium* oocysts are found mostly in feces of very young animals (1−3 weeks of age), with a lower incidence in older animals (de Graaf et al., 1999; Noordeen et al., 2001; Causape et al., 2002; Santín and Trout, 2007).

### 2.4 Pigs

The most common species and subtypes found in pigs are *C. parvum*, *C. suis*, and *C. scrofarum* formerly known as *Cryptosporidium* pig genotype II (Zahedi and Ryan, 2020), although *C. muris* and *C. tyzzeri* have also been reported occasionally (Robertson et al., 2014). As seen in ruminants, *Cryptosporidium* species tend to generally follow an age-related pattern: *C. suis* is more commonly found in piglets whereas starter pigs and fatteners primarily host *C. scrofarum* (Petersen et al., 2015).

There is a huge disparity in prevalence rates (0.1% to 100%) reported from all over the world (Robertson et al., 2014). Nonetheless, it is obvious that prevalence and intensity of infection is predominant in younger animals than older ones (Maddox-Hyttel et al., 2006; Petersen et al., 2015). While natural or experimental infections with the pig-adapted species, *C. suis* and *C. scrofarum*, are usually asymptomatic and cause mild or no illness (Robertson et al., 2014), experimental infection of piglets with either *C. parvum* or *C. hominis* results in watery diarrhea, anorexia, mucosal lesions, and increased mortality (Tzipori et al., 1994; Theodos et al., 1998; Lee et al., 2017).

## 3 Treatment options in humans and animals: The past and current state of affairs

Immunocompromised patients, neonatal animals, and young children especially malnourished ones are the most vulnerable to cryptosporidiosis, and hence, are the ones in most urgent need for effective therapeutics. Although cryptosporidiosis causes a self-limiting diarrheal illness in immunocompetent humans, patients do face a considerable risk for longer-term sequelae, especially in low-income countries. Additionally, *Cryptosporidium* infections in adult animals can lead to reduced production and result in economic losses to the livestock and food industry. As such, there is an urgent need for the development of safe, inexpensive, and efficacious drugs to reduce the ever-increasing worldwide burden of this disease. However, despite the widespread occurrence of the parasite, current effective treatment and prophylactic options for human and animal *Cryptosporidium* infections are virtually non-existent.

### 3.1 Humans

Several drugs with *in vitro* and *in vivo* anti-*Cryptosporidium* activity have been tested against human cases of cryptosporidiosis in uncontrolled/controlled clinical trials, open label/blinded studies, and case reports. These include macrolides, rifamycin derivatives, letrazuril, paromomycin, nitazoxanide, clofazimine, and other pharmacological agents (**Table 1**). In addition to drugs having direct anti-parasitic activity, other medications that augment the host immunity or ameliorate the symptoms/pathology of cryptosporidiosis have also been tested for the management of the disease (**Table 2**). However, unfortunately most of these treatments showed limited efficacy and inconsistent results when tested in the most susceptible target population including immunocompromised individuals and young children.

**Table 1:** Efficacies of treatments tested against cryptosporidiosis in human patients.

Drug	Age and health status of patients	Number of individuals	Study type	Reference	Treatment type	Efficacy	
						Clinical Cure	Parasitological Cure
Albendazole	Adults with advanced AIDS (CD4+ cell counts >200/mm^3^)	4	OL	(Zulu et al., 2002)	Therapeutic	+	+
Azithromycin	Adults with AIDS	85	R, DB, PC	(Soave et al., 1993)	Therapeutic	±	±
	Children on chemotherapy for cancer	2	CR	(Vargas et al., 1993)	Therapeutic	+	±
	Children with AIDS	4	CR	(Hicks et al., 1996)	Therapeutic	+	+
	Adults with AIDS	14	OL	(Blanshard et al., 1997)	Therapeutic	–	–
		13	OL, DC	(Dionisio et al., 1998)	Therapeutic	±	±
		54	RCR	(Holmberg et al., 1998)	Prophylactic	–	–
		41	R, OL, DC	(Kadappu et al., 2002)	Therapeutic	+	±
	Immunocompetent children	43	OL, AC	(Allam and Shehab, 2002)	Therapeutic	+	+
	Children on chemotherapy for cancer	2	CR	(Trad et al., 2003)	Therapeutic	+	+
Clarithromycin	Adults with AIDS	353	RCR	(Jordan, 1996)	Prophylactic	+	+
	Adults with AIDS	312	RCR	(Holmberg et al., 1998)	Prophylactic	+	+
	Adults with advanced AIDS	530	RCR	(Fichtenbaum et al., 2000)	Prophylactic	–	–
Clofazimine	Adults with advanced AIDS	20	R, DB, PC	(Iroh Tam et al., 2021)	Therapeutic	–	–
Diclazuril	Adults with AIDS	9	OL	(Connolly et al., 1990)	Therapeutic	–	–
		1	CR	(Menichetti et al., 1991)	Therapeutic	±	±
Letrazuril	Adults with advanced AIDS	1	CR	(Murdoch et al., 1993)	Therapeutic	+	+
		14	OL	(Harris et al., 1994)	Therapeutic	±	±
		35	OL	(Loeb et al., 1995)	Therapeutic	±	±
		10	OL	(Blanshard et al., 1997)	Therapeutic	±	±
Miltefosine	Malnourished adults with AIDS	7	OL	(Sinkala et al., 2011)	Therapeutic	±	–
Nitazoxanide	Adults with advanced AIDS	12	OL	(Doumbo et al., 1997)	Therapeutic	±	±
	Adults with AIDS	66	R, DB, PC	(Rossignol et al., 1998)	Therapeutic	±	±
	Immunocompetent adults and children	99	R, DB, PC	(Rossignol et al., 2001)	Therapeutic	+	±
	Malnourished HIV-seronegative children	47	R, DB, PC	(Amadi et al., 2002)	Therapeutic	±	±
	Malnourished HIV-seropositive children	49				–	–
	Adults with AIDS	207	R, DB, PC	(Zulu et al., 2005)	Therapeutic	–	–
	Immunocompetent adults and adolescents	86	R, DB, PC	(Rossignol et al., 2006)	Therapeutic	+	+
	Children and adults with AIDS	357	CU, OL	(Rossignol, 2006)	Therapeutic	±	±
	Children with AIDS	3	CU, CR	(Abraham et al., 2008)	Therapeutic	+	+
	Children with AIDS	52	R, DB, PC	(Amadi et al., 2009)	Therapeutic	–	–
	Pediatric solid organ transplant recipients	6	RCR	(Krause et al., 2012)	Therapeutic	+	+
	Immunocompetent children	135	R, OL, AC, PC	(Hussien et al., 2013)	Therapeutic	+	+
	Immunocompetent adults	58	OL	(Ali et al., 2014)	Therapeutic	±	±
	Adult renal transplant recipients	13	RCR	(Bhadauria et al., 2015)	Therapeutic	±	±
	Immunocompetent children	60	R, DB, PC	(Abaza et al., 2016)	Therapeutic	+	+
	Immunocompromised children	60				±	±
	Adults on chemotherapy for cancer	2	CR	(Demonchy et al., 2021)	Therapeutic	±	±
Paromomycin	Adults with AIDS	12	RCR	(Gathe et al., 1990)	Therapeutic	±	±
		5	CR	(Clezy et al., 1991)	Therapeutic	±	±
		5	CR	(Armitage et al., 1992)	Therapeutic	±	±
		1	CR	(Danziger et al., 1993)	Therapeutic	±	±
		7	RCR	(Fichtenbaum et al., 1993)	Therapeutic	±	±
		6	CR	(Wallace et al., 1993)	Therapeutic	±	±
		24	OL	(Bissuel et al., 1994)	Therapeutic	±	±
		35	OL	(Scaglia et al., 1994)	Therapeutic	±	±
		10	R, DB, PC	(White et al., 1994)	Therapeutic	+	+
		44	OL	(Flanigan et al., 1996)	Therapeutic	±	±
		20	OL	(Blanshard et al., 1997)	Therapeutic	±	±
		70	RCR	(Hashmey et al., 1997)	Therapeutic	±	±
		35	R, DB, PC	(Hewitt et al., 2000)	Therapeutic	–	–
	Children on chemotherapy for cancer	3	CR	(Trad et al., 2003)	Therapeutic	+	±
	Immunocompetent children	38	OL	(Vandenberg et al., 2012)	Therapeutic	±	±
		135	R, OL, AC, PC	(Hussien et al., 2013)	Therapeutic	±	±
Roxithromycin	Adults with AIDS	24	OL	(Sprinz et al., 1998)	Therapeutic	+	±
		22	OL	(Uip et al., 1998)	Therapeutic	+	±
Rifabutin	Adults with AIDS	214	RCR	(Holmberg et al., 1998)	Prophylactic	+	+
	Adults with advanced AIDS	650	RCR	(Fichtenbaum et al., 2000)	Prophylactic	+	+
Rifaximin	Adults and children infected with HIV (CD4+ cell counts >200/mm^3^)	10	OL	(Amenta et al., 1999)	Therapeutic	+	+
	Adult solid organ transplant recipient	1	CR	(Burdese et al., 2005)	Therapeutic	+	+
	Adults with AIDS (CD4+ cell counts <50 cells/mm^3^)	5	CR	(Gathe et al., 2008)	Therapeutic	+	+
Spiramycin	Adult with AIDS	1	N of 1 trial	(Woolf et al., 1987)	Therapeutic	–	–
	Immunocompromised adults	37	CU, OL	(Moskovitz et al., 1988)	Therapeutic	+	±
	Immunocompetent infants	44	DB, PC	(Saez-Llorens et al., 1989)	Therapeutic	+	+
	Malnourished infants	39	R, DB, PC	(Wittenberg et al., 1989)	Therapeutic	–	–
	Adults with AIDS	31	CR	(Weikel et al., 1991)	Therapeutic	–	–

1. AC, active-controlled; DB, double-blind; CU, compassionate use; CR, case report; DC, dose comparison; OL, open-label; PC, placebo-controlled; R, randomized; RCR, retrospective case review.

2. “+” = complete resolution; “-” = no demonstrable activity; “±” = partial resolution or relapse after treatment discontinuation.

**Table 2:** Other immunological and supportive treatments tested for efficacy against cryptosporidiosis in human patients.

Drug	Age and health status of patients	Number of individuals	Study type	Reference	Treatment type	Efficacy	
						Clinical Cure	Parasitological Cure
Bovine leukocyte extract	Adults and a child with AIDS	8	CR	(Louie et al., 1987)	Therapeutic	±	±
	Adults with AIDS	14	R, DB, PC	(McMeeking et al., 1990)	Therapeutic	+	+
Human serum immune globulin	Child on chemotherapy for cancer	1	CR	(Borowitz and Saulsbury, 1991)	Therapeutic	+	+
Hyperimmune bovine colostrum	Immunocompromised children and adult	3	CR	(Tzipori et al., 1987)	Therapeutic	+	±
	Adults with AIDS	5	R, DB, PC	(Nord et al., 1990)	Therapeutic	±	±
		1	CR	(Ungar et al., 1990)	Therapeutic	+	+
	Immunodeficient children and adults	7	OL	(Rump et al., 1992)	Therapeutic	+	+
	Adults with AIDS	7	OL	(Plettenberg et al., 1993)	Therapeutic	±	±
	Child infected with HIV	1	CR	(Shield et al., 1993)	Therapeutic	+	+
	Adults with AIDS	20	OL	(Greenberg and Cello, 1996)	Therapeutic	±	–
	Immunocompetent adults	16	R, DB, PC	(Okhuysen et al., 1998)	Prophylactic	±	±
	Adults with AIDS	20	OL	(Floren et al., 2006)	Therapeutic	+	NR
Somatostatin analogs (octreotide and vapreotide)	Adults with AIDS	1	CR	(Cook et al., 1988)	Therapeutic	+	–
		1	CR	(Katz et al., 1988)	Therapeutic	+	–
		4	CR	(Clotet et al., 1989)	Therapeutic	±	–
		15	OL	(Cello et al., 1991)	Therapeutic	±	–
		18	OL	(Romeu et al., 1991)	Therapeutic	±	–
		21	OL	(Girard et al., 1992)	Therapeutic	±	–
		4	OL	(Liberti et al., 1992)	Therapeutic	±	–
		13	OL	(Moroni et al., 1993)	Therapeutic	±	–
HIV protease inhibitor (indinavir or saquinavir)	Adults with advanced AIDS (CD4+ count < 50/mm^3^)	1	CR	(Grube et al., 1997)	Therapeutic	+	+
		5	OL	(Bobin et al., 1998)	Therapeutic	+	+
		2	R, OL	(Foudraine et al., 1998)	Therapeutic	+	+
HAART including HIV protease inhibitor	Adults with AIDS (CD4+ count < 400/mm^3^)	4	OL	(Carr et al., 1998)	Therapeutic	+	+
HAART including HIV protease inhibitor	Adults with advanced AIDS (CD4+ count < 50/mm^3^)	3	CR	(Miao et al., 1999; Miao et al., 2000)	Therapeutic	+	+

1. DB, double-blind; CR, case report; NR, not reported; OL, open-label; PC, placebo-controlled; R, randomized.

2. “+” = complete resolution; “-” = no demonstrable benefit; “±” = partial resolution or relapse after treatment discontinuation.

#### 3.1.1 Nitazoxanide

Thus far, nitazoxanide is the only drug approved by the United States Food and Drug Administration (FDA) for the treatment of cryptosporidiosis in immunocompetent human patients (Checkley et al., 2015). Nitazoxanide is a member of the thiazole class of drugs that was initially developed as a veterinary anthelmintic but was later reported to have broad-spectrum activity against parasites, viruses, and bacteria. This drug acts by inhibiting the pyruvate:ferredoxin/flavodoxin oxidoreductase (PFOR), an enzyme essential for the anaerobic energy metabolism of various microorganisms (Hoffman et al., 2007). However, the exact mechanism of action against *Cryptosporidium* remains questionable since these parasites encode a unique PFOR with a fused C-terminal cytochrome P450 domain (Rotte et al., 2001). Interestingly, nitazoxanide was shown to inhibit the growth of *C. parvum* by more than 90% at a concentration of 10 µg/ml (32 µM) in cell culture but was ineffective in the anti-IFN-γ-conditioned SCID mouse model of cryptosporidiosis even at high doses (Theodos et al., 1998). Furthermore, nitazoxanide has also been found to be ineffective in other immunodeficient or immunocompromised animal models of cryptosporidiosis, questioning the true efficacy of the drug (Lee et al., 2017; Jumani et al., 2018).

Various randomized placebo-controlled studies have found nitazoxanide to be helpful in treating cryptosporidiosis in adults and children without HIV resulting in reduced duration of both diarrhea and oocyst shedding (Rossignol et al., 2001; Amadi et al., 2002; Rossignol et al., 2006; Hussien et al., 2013; Abaza et al., 2016). Studies conducted in Egyptian immunocompetent adults and children demonstrated significantly higher clinical and parasitological cure rates compared with the placebo-treated groups (Rossignol et al., 2001; Rossignol et al., 2006). In a randomized controlled trial involving malnourished children in Zambia, nitazoxanide treatment for 3 days yielded a partial but significantly better cure than placebo (Amadi et al., 2002). Recent controlled trials have also reported complete clinical and parasitological recovery in most immunocompetent children with cryptosporidiosis (Hussien et al., 2013; Abaza et al., 2016).

However, a meta-analysis of seven randomized controlled trials involving 169 participants with cryptosporidiosis confirmed the absence of obvious evidence of efficacy of nitazoxanide in HIV-seropositive patients (Abubakar et al., 2007). A course of nitazoxanide does not appear to improve the resolution of diarrhea and parasitological outcome in HIV-infected and immunocompromised patients (Doumbo et al., 1997; Rossignol et al., 1998; Amadi et al., 2002; Zulu et al., 2005; Amadi et al., 2009; Abaza et al., 2016). In the first randomized controlled trial of this drug in adult HIV patients with cryptosporidiosis, better overall parasite clearance rates were seen in the treated group compared with the placebo one, but significant differences were only seen in those with CD4+ T-cell counts above 50/mm^3^ (Rossignol et al., 1998). Other randomized placebo-controlled trials conducted by Amadi and others in HIV-positive children in Zambia have also documented no beneficial effect of nitazoxanide over placebo in terms of clinical and parasitological cure rates or mortality (Amadi et al., 2002; Amadi et al., 2009). Moreover, only moderate efficacy was achieved in a study of cryptosporidiosis in immunocompromised children even after prolonged nitazoxanide treatment of up to 28 days (Abaza et al., 2016). While prolonged therapy with higher doses of the drug is somewhat effective in treating cryptosporidiosis in patients with compromised immunity, normal prescribed doses and short-term duration of therapy are inadequate for preventing recurrence of disease symptoms after treatment discontinuation (Abraham et al., 2008; Krause et al., 2012; Ali et al., 2014; Bhadauria et al., 2015; Demonchy et al., 2021). Therefore, it is evident that nitazoxanide therapy is clearly futile in treating cryptosporidiosis in advanced AIDS patients and other severely immunocompromised patients.

Lack of efficacy in immunocompromised animal models and humans suggests that a healthy host immune system is essential to the effectiveness of nitazoxanide. Nitazoxanide treatment has been recently shown to result in broad amplification of the host cell innate immune response to viral infections, including an increase in interferon activities (Jasenosky et al., 2019). If an immune defect renders the host incapable of generating an interferon-γ-dependent response, nitazoxanide would be expected to be ineffective in such immunocompromised hosts (Theodos et al., 1998; Jumani et al., 2018), given the importance of these innate responses in controlling *Cryptosporidium* at its initial stages of infection (McDonald et al., 2013). Similarly, lack of curative effect of nitazoxanide in advanced AIDS patients (with low CD4+ T-cell counts) suffering from chronic cryptosporidiosis can be explained by the fact that adaptive immunity plays a crucial role in clearing the parasites completely from the infected host (Mead, 2014). Thus, the efficacy of nitazoxanide seems to be closely related to both the innate and adaptive immune status of the host.

#### 3.1.2 Paromomycin

Another well studied drug, a poorly absorbed aminoglycoside paromomycin, has been investigated against cryptosporidiosis in three published controlled trials (White et al., 1994; Hewitt et al., 2000; Hussien et al., 2013), but results have been highly divergent and mostly discouraging. Paromomycin, like other aminoglycoside antibiotics, inhibits protein synthesis by binding to the 30S ribosomal subunit and shows broad spectrum activity against bacteria and some protozoa (Lin et al., 2018). Several uncontrolled trials and case studies in AIDS patients suffering from cryptosporidiosis have reported favorable clinical outcomes after paromomycin treatment (Gathe et al., 1990; Clezy et al., 1991; Armitage et al., 1992; Danziger et al., 1993; Fichtenbaum et al., 1993; Wallace et al., 1993; Bissuel et al., 1994; Scaglia et al., 1994; Hashmey et al., 1997). However, the patient responses in most cases were short-lived and continuous maintenance therapy was required to prevent frequent relapses after treatment discontinuation suggesting that complete parasitological cure was not achieved in these cases. Children suffering from cryptosporidiosis have been reported to respond favorably after treatment with paromomycin, although the overall clinical and parasitological response is reduced as compared to nitazoxanide or azithromycin (Trad et al., 2003; Vandenberg et al., 2012; Hussien et al., 2013).

#### 3.1.3 Macrolides

Macrolides are a class of antibiotics that disrupt bacterial protein synthesis by binding to the 50S subunit of the ribosome (Lin et al., 2018). Among the macrolides tested for efficacy against human cryptosporidiosis, azithromycin remains the most studied. In a multi-center, placebo-controlled, double-blind study, preliminary data analysis revealed no significant improvement in clinical symptoms and oocyst numbers in azithromycin-treated AIDS patients with cryptosporidiosis (Soave et al., 1993). Interestingly, however, a statistically significant decrease in cryptosporidial oocyst shedding was reported in patients with appropriate azithromycin serum concentrations (Soave et al., 1993). By contrast, azithromycin was found to have no therapeutic or prophylactic efficacy in the management of cryptosporidial diarrhea in AIDS patients (Blanshard et al., 1997; Holmberg et al., 1998). While short-term azithromycin treatment for cryptosporidiosis was unable to achieve total parasitological clearance and prevent relapses in AIDS patients, long-term and low dose maintenance therapy was associated with noticeable clinical and parasitological benefits (Dionisio et al., 1998; Kadappu et al., 2002). Nevertheless, azithromycin seems to be more effective in treating children with cryptosporidiosis. Prompt clinical improvement and high parasite clearance rates have been reported after azithromycin therapy in both immunocompetent and immunocompromised children (Vargas et al., 1993; Hicks et al., 1996; Allam and Shehab, 2002; Trad et al., 2003).

Clarithromycin has been tested in humans with AIDS for prophylactic effectiveness against cryptosporidiosis. But studies have reported conflicting results with some indicating a highly protective effect against the development of cryptosporidiosis (Jordan, 1996; Holmberg et al., 1998) while others concluding that the drug is not useful in preventing cryptosporidiosis in this patient population (Fichtenbaum et al., 2000). Similarly, spiramycin has also shown inconsistent results in treating cryptosporidiosis in controlled and uncontrolled trials involving infants (Saez-Llorens et al., 1989; Wittenberg et al., 1989) and adult patients (Woolf et al., 1987; Moskovitz et al., 1988) with reports of acute intestinal injury in some patients receiving high doses of the drug (Weikel et al., 1991). Another macrolide, roxithromycin has proven effective in uncontrolled studies of AIDS patients with cryptosporidial enteritis. However, complete parasite clearance was only achieved in half of the treated patients and the results were not compared with a placebo-treated control group (Sprinz et al., 1998; Uip et al., 1998).

#### 3.1.4 Rifamycin derivatives

Rifamycins are a group of drugs that are highly active against mycobacterial infections. Members of this antibiotic class inhibit RNA synthesis by selective binding of the bacterial DNA-dependent RNA polymerase (Hartmann et al., 1967). Several uncontrolled studies have evaluated rifamycin derivatives, namely rifabutin and rifaximin, for prophylactic and therapeutic efficacy respectively, against cryptosporidiosis in HIV-infected humans. Rifabutin has been found to be highly effective in preventing the development of cryptosporidiosis in AIDS patients receiving chemoprophylaxis for *Mycobacterium avium* complex infection (Holmberg et al., 1998; Fichtenbaum et al., 2000). Similarly, some studies have demonstrated a significant clinical and parasitological benefit of rifaximin, a poorly absorbed rifamycin, in the treatment of cryptosporidiosis in solid organ transplant recipient patients (Burdese et al., 2005) and a small number of HIV-infected adults and children with CD4+ T-cell counts ranging from <50 cells/mm^3^ to >200 cells/mm^3^ (Amenta et al., 1999; Gathe et al., 2008). Thus, these results warrant the testing of these drugs in larger randomized controlled clinical trials for confirmation of anti-*Cryptosporidium* efficacy.

#### 3.1.5 Benzene acetonitrile derivatives

Diclazuril and letrazuril have been both shown to be active against *Eimeria* species, parasites closely related to *Cryptosporidium*, although the exact mode of action of these drugs is currently unknown. Yet, diclazuril failed to show any obvious effect in severe cryptosporidiosis in adults with HIV infection (Connolly et al., 1990). In another study, a single patient showed both clinical and parasitological response to diclazuril treatment but the infection was less severe and the patient also received antiretroviral therapy during and after the treatment course (Menichetti et al., 1991). Additionally, letrazuril, the p-fluor analog of diclazuril, shows only partial efficacy against advanced AIDS-related cryptosporidial diarrhea (Harris et al., 1994; Loeb et al., 1995; Blanshard et al., 1997) and treated patients develop temporary drug-related side-effects including abnormal liver function tests and skin rashes that tend to resolve after treatment discontinuation (Murdoch et al., 1993; Harris et al., 1994; Loeb et al., 1995).

#### 3.1.6 Miscellaneous antimicrobials

##### 3.1.6.1 Clofazimine

Clofazimine, an FDA-approved antimycobacterial drug primarily used to treat leprosy, was found to be effective against both *C*. *parvum* and *C*. *hominis in vitro* and showed promising efficacy against *C*. *parvum* in a mouse model (Love et al., 2017). Interestingly, the mechanism of action of clofazimine as an anti-mycobacterial drug is not well understood. A recent randomized controlled clinical trial tested the efficacy of the drug in enrolled patients with advanced HIV infection and *Cryptosporidium*-associated diarrhea. The findings of the study, however, failed to demonstrate any effectiveness of clofazimine in reducing fecal parasite shedding and stool frequency in immunocompromised patients (Iroh Tam et al., 2021). Moreover, unexpected adverse events were higher in the clofazimine-treated patients as compared to the placebo control group.

##### 3.1.6.2 Miltefosine

Like clofazimine, miltefosine an anti-*Leishmania* drug, has also shown promising *in vitro* efficacy against *C. parvum* as per anecdotal observations but its specific mode of action is not entirely known. This drug, however, showed modest clinical improvement without evidence of oocyst clearance in treated HIV-infected malnourished individuals (Sinkala et al., 2011). Furthermore, adverse events including hepatic dysfunction and renal failure were observed in some patients, leading to premature termination of the phase-1–phase-2 trial.

##### 3.1.6.3 Albendazole

Albendazole is a broad-spectrum anthelmintic drug that is known to bind to β-tubulin and inhibit microtubule assembly in helminth worms (Lacey, 1988). This benzimidazole derivative was shown to have a significant effect on the duration of diarrhea in a randomized, controlled trial in HIV-seropositive patients with persistent diarrhea (Kelly et al., 1996). Later, another study assessed the effect of albendazole on *C. parvum* and other intracellular protozoa including *Isospora* and microsporidia in HIV-positive patients and found the drug to exhibit complete *C*. *parvum* clearance when used at doses higher than the normal prescribed dose (Zulu et al., 2002). However, the number of HIV patients with cryptosporidiosis in this study was very small as compared to patients infected with other protozoa, and the results cannot be considered credible due to the lack of an untreated control group for comparison. Nevertheless, it does seem that albendazole has some activity against *Cryptosporidium* at high doses (Fayer and Fetterer, 1995), but, to the best of our knowledge, no other study has evaluated albendazole for anti-*Cryptosporidium* efficacy in immunocompromised humans.

#### 3.1.7 Other treatments

Although most studies on the treatment of cryptosporidiosis have been carried out by repurposing the use of antibacterial drugs, some studies have tested other treatments with alternate modes of action such as immune cell extracts, immunoglobulins, hyperimmune colostrum, somatostatin analogs, and highly active antiretroviral therapy (HAART) (**Table 2**).

##### 3.1.7.1 Immunotherapy

Passive immunotherapy by oral administration of immunoglobulins derived from bovine colostrum or human serum was shown to be effective in *Cryptosporidium*-infected immunosuppressed humans in several open-label uncontrolled studies and case reports (Tzipori et al., 1987; Ungar et al., 1990; Borowitz and Saulsbury, 1991; Rump et al., 1992; Shield et al., 1993; Floren et al., 2006), but results obtained from randomized double-blind controlled studies have been disappointing (Nord et al., 1990; Okhuysen et al., 1998). Similarly, treatment of AIDS-associated cryptosporidial diarrhea by oral administration of cellular extracts prepared from lymphocytes obtained from immunized calves produced mixed results (Louie et al., 1987; McMeeking et al., 1990).

##### 3.1.7.2 Somatostatin analogs

Somatostatin analogs including octreotide and vapreotide have been reported to improve secretory diarrhea by inhibiting the motility and secretions of the gastro-intestinal tract. Patients with AIDS-related chronic diarrhea, especially those without specific pathogens, may benefit from treatment with this class of drugs. However, these agents have mostly been ineffective or partially effective in reducing the fecal output in *Cryptosporidium*-associated diarrhea in HIV-infected patients and show no parasitological cure (Cook et al., 1988; Katz et al., 1988; Clotet et al., 1989; Cello et al., 1991; Romeu et al., 1991; Girard et al., 1992; Liberti et al., 1992; Moroni et al., 1993).

##### 3.1.7.3 Highly active antiretroviral therapy

Cryptosporidiosis is typically a self-limiting illness in immunocompetent individuals, and therefore, immune reconstitution by restoring CD4+ T-cell levels in particular, is an essential part of the disease management strategy in the immunocompromised (Flanigan et al., 1992). HIV-infected patients with CD4+ T-cell counts below 200/mm^3^ tend to be susceptible to a higher frequency of cryptosporidial infections highlighting the relationship of these opportunistic pathogens with the immune status of an individual (de Oliveira-Silva et al., 2007; Tuli et al., 2008; Adamu et al., 2013; Nsagha et al., 2016; Cerveja et al., 2017; Amoo et al., 2018). The use of HAART in HIV-infected patients has significantly reduced the global frequency and severity of cryptosporidiosis in this patient population (Le Moing et al., 1998; Call et al., 2000; Conti et al., 2000; Ives et al., 2001; Babiker et al., 2002; Bachur et al., 2008; Missaye et al., 2013). HAART re-establishes CD4+ T-cell counts and inhibits viral replication using a combination of nucleoside reverse transcriptase inhibitors (NRTIs), non-nucleoside reverse transcriptase inhibitors (NNRTIs), and HIV protease inhibitors. However, chronic diarrhea at initiation of HAART in HIV-positive patients has been associated with increased early mortality, emphasizing the need for early anti-retroviral therapy before the onset of diarrhea (Dillingham et al., 2009). Most studies involving HAART for the treatment of cryptosporidiosis have used HIV protease inhibitors either individually or in combination with other antiretrovirals to successfully treat the disease with major clinical and parasitological benefits (Grube et al., 1997; Bobin et al., 1998; Carr et al., 1998; Foudraine et al., 1998; Miao et al., 1999; Miao et al., 2000). Such a therapy may exert its pharmacological effect against AIDS-associated cryptosporidiosis both by restoration of circulating CD4+ T-cell counts and direct inhibition of *Cryptosporidium* proteases (Mele et al., 2003; Pantenburg et al., 2009). But individuals with other causes of weakened immunity, including primary immunodeficiency, immunosuppressive therapy in organ transplant recipients, and chemotherapy in cancer patients, remain at high risk of severe cryptosporidiosis.

#### 3.1.8 Combination therapy

Several combination therapies involving the use of either nitazoxanide or paromomycin in conjunction with macrolides, rifamycin derivatives, or HAART have shown promising efficacy for cryptosporidiosis in small uncontrolled trials and case studies with both clinical improvement and parasite elimination in a range of affected immunocompromised individuals (**Table 3**). However, these results need to be replicated in large, controlled trials before any definite conclusions can be drawn regarding the efficacy of such combinations. Huang and colleagues conducted a randomized placebo-controlled trial to investigate the therapeutic effects of acetylated spiramycin and garlicin on *Cryptosporidium* infection in institutionalized drug users. Although the combination treatment achieved high parasitological cure rates, this study was carried out in asymptomatic *Cryptosporidium* carriers without ascertaining the HIV/immune status of the enrolled individuals, and therefore has limited clinical significance (Huang et al., 2015).

**Table 3:** Efficacies of various combination therapies tested against human cryptosporidiosis.

Drug combination	Age and health status of patients	Number of individuals	Study type	Reference	Treatment type	Efficacy	
						Clinical Cure	Parasitological Cure
Acetylspiramycin + garlicin	Asymptomatic adult drug users	151	R, PC	(Huang et al., 2015)	Therapeutic	–	+
Azithromycin + paromomycin	Adults with AIDS	11	OL	(Smith et al., 1998)	Therapeutic	+	±
	Adult with AIDS	1	CR	(Palmieri et al., 2005)	Therapeutic	+	+
	Adult with AIDS	1	CR	(Meamar et al., 2006)	Therapeutic	+	+
	Adult liver transplant recipient	1	CR	(Denkinger et al., 2008)	Therapeutic	+	+
Azithromycin + paromomycin + nitazoxanide	Pediatric renal transplant recipient	1	CR	(Hong et al., 2007)	Therapeutic	+	+
Azithromycin + nitazoxanide	Adult allogeneic hematopoietic stem cell transplant recipients	5	OL	(Legrand et al., 2011)	Therapeutic	+	+
	Child on chemotherapy for cancer	1	CR	(Bakliwal et al., 2021)	Therapeutic	+	+
	Immunosuppressed child with CD40L deficiency	1	CR	(Dupuy et al., 2021)	Therapeutic	+	+
Azithromycin + nitazoxanide + rifaximin	Adult renal transplant recipient	1	CR	(Tomczak et al., 2022)	Therapeutic	+	+
Clarithromycin + rifabutin	Adults with advanced AIDS	451	RCR	(Fichtenbaum et al., 2000)	Prophylactic	±	±
Antiretrovirals + Paromomycin, Spiramycin, or Azithromycin	Adults with AIDS (CD4+ count < 180/mm^3^)	45	RCR	(Maggi et al., 2000)	Therapeutic	+	+
HAART + Paromomycin	Adults with advanced AIDS (CD4+ count < 50/mm^3^)	1	CR	(Schmidt et al., 2001)	Therapeutic	+	+
HAART + Glutamine + Azithromycin + Paromomycin		1	CR	(Moling et al., 2005)	Therapeutic	+	±
Nitazoxanide + fluoroquinolone	Adult renal transplant recipients	21	RCR	(Bhadauria et al., 2015)	Therapeutic	+	+
Spiramycin + paromomycin + nitazoxanide	Pediatric renal transplant recipient	1	CR	(Acikgoz et al., 2012)	Therapeutic	+	+

1. DB, double-blind; CR, case report; OL, open-label; PC, placebo-controlled; R, randomized; RCR, retrospective case review.

2. “+” = complete resolution; “-” = no demonstrable activity; “±” = partial resolution or relapse after treatment discontinuation.

Certain clinical case reports have documented favorable clinical and parasitological outcomes in HIV-infected and organ transplant recipient patients diagnosed with extra-intestinal and intestinal cryptosporidiosis, after antimicrobial combination therapy with azithromycin and paromomycin (Palmieri et al., 2005; Meamar et al., 2006; Denkinger et al., 2008). In a small open-label, uncontrolled study, patients with AIDS (<100 CD4+ T-cells/mm^3^) and chronic cryptosporidiosis, showed a marked improvement in stool frequency and a significant decrease in fecal excretion of *Cryptosporidium* oocysts in response to azithromycin/paromomycin combination therapy (Smith et al., 1998). However, follow-up study after the completion of treatment revealed the persistence of chronic, mild diarrhea in some patients.

Complete resolution of diarrhea as well as elimination of the parasite has been reported in immunosuppressed children and adults suffering from cryptosporidiosis after dual therapy with azithromycin and nitazoxanide (Legrand et al., 2011; Bakliwal et al., 2021; Dupuy et al., 2021). Additionally, triple therapy involving azithromycin, nitazoxanide, and paromomycin or rifaximin led to complete clinical and parasitological cure with no relapse in renal transplant patients (Hong et al., 2007; Tomczak et al., 2022). Another study successfully treated cryptosporidiosis in a pediatric renal transplant patient using a triple therapy consisting of spiramycin, nitazoxanide, and paromomycin (Acikgoz et al., 2012).

Moreover, quite a few small uncontrolled studies suggest that a combination of antimicrobials and HAART (especially with protease inhibitors) dramatically accelerates the clinical response in AIDS patients suffering from cryptosporidiosis (Maggi et al., 2000; Schmidt et al., 2001; Moling et al., 2005). But data from randomized controlled trials is required to support these results given the self-limiting nature of the disease. Nevertheless, increasing evidence has demonstrated that combination therapy achieves better clinical and microbiological resolution rates than monotherapy for the treatment of cryptosporidiosis in immunocompromised patients (Maggi et al., 2000; Bhadauria et al., 2015; Lanternier et al., 2017; Bakliwal et al., 2021; Tomczak et al., 2022).

### 3.2 Animals

Numerous antimicrobial compounds have been screened and evaluated for efficacy against naturally acquired and experimentally induced cryptosporidiosis in animals (**Table 4**), albeit with limited success. Most of the tested drugs exhibit only partial prophylactic and therapeutic efficacy in reducing oocyst excretion and disease severity in affected animals. Thus far, no effective currently licensed therapeutics are available in the United States for *Cryptosporidium* infections in animals (Santin, 2020; Zahedi and Ryan, 2020). Alternatively, several supportive and immunological therapies have also been tested for the management of cryptosporidiosis in livestock (**Table 5**), but none have shown promise in changing the course of the disease.

**Table 4:** Anti-cryptosporidial efficacies of various antimicrobial and novel treatments in farm animals and natural host animal models.

Therapeutic agent	Animal species and age	Number of individuals	Type of infection	Reference	Treatment type	Efficacy	
						Clinical Cure	Parasitological Cure
Aminoacyl-tRNA synthetase inhibitor Compound 2093	Neonatal calves	6	Experimental	(Hasan et al., 2021)	Therapeutic	±	±
Azithromycin	Neonatal calves	50	Natural	(Elitok et al., 2005)	Therapeutic	+	±
		25	Natural	(Nasir et al., 2013)	Therapeutic	NR	±
	Gnotobiotic piglets	40	Experimental	(Lee et al., 2017)	Therapeutic	±	–
	Buffalo calf	1	Natural	(Maurya et al., 2016)	Therapeutic	+	NR
Benzoxaborole AN7973	Neonatal calves	17	Experimental	(Lunde et al., 2019)	Therapeutic	+	+
Bumped Kinase Inhibitor 1294	Neonatal calves	18	Experimental	(Lendner et al., 2015)	Prophylactic	±	±
		24	Experimental	(Schaefer et al., 2016)	Therapeutic	±	+
Bumped Kinase Inhibitor 1369	Neonatal calves	7	Experimental	(Hulverson et al., 2017)	Therapeutic	+	+
	Gnotobiotic piglets	18	Experimental	(Lee et al., 2018)	Therapeutic	+	+
Decoquinate	Neonatal calves	30	Experimental	(Redman and Fox, 1993)	Prophylactic	±	±
		43	Experimental	(Moore et al., 2003)	Prophylactic	–	–
		90	Natural	(Lallemand et al., 2006)	Prophylactic	–	–
	Neonatal goat kids	20	Experimental	(Mancassola et al., 1997)	Prophylactic	+	±
		64	Natural	(Ferre et al., 2005)	Prophylactic	±	±
Halofuginone lactate	Neonatal calves	150	Natural	(Villacorta et al., 1991)	Prophylactic	+	±
		20	Experimental	(Naciri et al., 1993)	Prophylactic	+	±
		70	Natural and experimental	(Peeters et al., 1993)	Prophylactic	+	±
		158	Natural	(Lefay et al., 2001)	Prophylactic	+	±
		152	Natural	(Joachim et al., 2003)	Metaphylactic	±	±
		31	Natural	(Jarvie et al., 2005)	Prophylactic	±	±
		90	Natural	(Lallemand et al., 2006)	Prophylactic	–	±
		260	Natural	(Klein, 2008)	Prophylactic	±	±
					Therapeutic	±	±
		32	Natural	(De Waele et al., 2010)	Prophylactic	+	±
		513	Natural	(Trotz-Williams et al., 2011)	Prophylactic	±	±
		45	Natural	(Almawly et al., 2013)	Prophylactic	–	–
		149	Natural	(Keidel and Daugschies, 2013)	Metaphylactic	±	±
		530	Natural	(Meganck et al., 2015)	Prophylactic	±	±
		144	Natural	(Niine et al., 2018)	Prophylactic	±	–
		20	Natural	(Aydogdu et al., 2018)	Therapeutic	±	±
		123	Natural	(Velez et al., 2019)	Prophylactic	–	±
	Neonatal lambs	12	Experimental	(Naciri and Yvore, 1989)	Prophylactic	+	+
		5			Therapeutic	+	±
		28	Natural	(Causapé et al., 1999)	Prophylactic	–	±
					Therapeutic	±	±
		1170	Natural	(Giadinis et al., 2007)	Prophylactic	+	±
					Therapeutic	+	+
	Neonatal goat kids	69	Natural	(Chartier et al., 1999)	Prophylactic	±	±
		2240	Natural	(Giadinis et al., 2008)	Prophylactic	+	+
					Therapeutic	+	+
		44	Experimental	(Petermann et al., 2014)	Prophylactic	±	±
Lasalocid	Neonatal calves	10	Experimental	(Moon et al., 1982)	Prophylactic	±	±
		11	Natural	(Sahal et al., 2005)	Therapeutic	+	±
		12	Natural	(Murakoshi et al., 2014)	Prophylactic	+	±
Nitazoxanide	Neonatal calves	20	Experimental	(Ollivett et al., 2009)	Therapeutic	+	±
		9	Experimental	(Schnyder et al., 2009)	Prophylactic	–	–
					Therapeutic	–	–
	Neonatal goat kids	47	Experimental	(Viel et al., 2007)	Prophylactic	NR	±
	Gnotobiotic piglets	31	Experimental	(Theodos et al., 1998)	Therapeutic	–	±
		40	Experimental	(Lee et al., 2017)	Therapeutic	±	±
Paromomycin	Neonatal calves	16	Experimental	(Fayer and Ellis, 1993)	Prophylactic	+	+
		20	Natural	(Grinberg et al., 2002)	Prophylactic	±	±
		20	Natural	(Aydogdu et al., 2018)	Therapeutic	±	±
	Neonatal goat kids	19	Experimental	(Mancassola et al., 1995)	Prophylactic	+	+
		30	Natural	(Chartier et al., 1996)	Prophylactic	+	±
		55	Natural	(Johnson et al., 2000)	Prophylactic	+	+
	Neonatal lambs	36	Natural	(Viu et al., 2000)	Therapeutic	±	+
	Gnotobiotic piglets	21	Experimental	(Tzipori et al., 1994)	Therapeutic	±	±
		31	Experimental	(Theodos et al., 1998)	Therapeutic	+	±
Triazolopyradizine MMV665917	Neonatal calves	13	Experimental	(Stebbins et al., 2018)	Therapeutic	+	+
	Gnotobiotic piglets	28	Experimental	(Lee et al., 2019)	Therapeutic	+	±
Pyrazolopyridine KDU731	Neonatal calves	13	Experimental	(Manjunatha et al., 2017)	Therapeutic	+	+
Sulfonamides	Neonatal calves	59	Natural	(Fischer, 1983)	Prophylactic	–	–
					Therapeutic	–	–
		13	Experimental	(Fayer, 1992)	Prophylactic	–	–
		152	Natural	(Joachim et al., 2003)	Metaphylactic	–	–
		25	Natural	(Nasir et al., 2013)	Therapeutic	NR	–
	Neonatal goat kids	24	Experimental	(Koudela and Bokova, 1997)	Prophylactic	–	–
					Therapeutic	–	–
Tilmicosin	Neonatal goat kids	22	Natural	(Paraud et al., 2010)	Prophylactic	–	–
Tylosin	Neonatal calves	23	Natural	(Duru et al., 2013)	Therapeutic	+	±

1. NR, not reported.

2. “+” = complete cure; “-” = no demonstrable effect; “±” = partial cure or relapse after treatment discontinuation.

**Table 5:** Alternate treatments tested for efficacy against cryptosporidiosis in farm animals and natural host animal models.

Drug	Animal species and age	Number of individuals	Type of infection	Study	Treatment type	Efficacy	
						Clinical Cure	Parasitological Cure
α-cyclodextrin	Neonatal goat kids	20	Experimental	(Castro-Hermida et al., 2004)	Prophylactic	±	±
β-cyclodextrin	Neonatal calves	12	Natural	(Castro-Hermida et al., 2001a)	Prophylactic	+	±
					Therapeutic	±	±
	Neonatal lambs	53	Natural	(Castro-Hermida et al., 2001b)	Prophylactic	+	+
					Therapeutic	+	±
Activated charcoal	Neonatal calves	258	Natural	(Ross et al., 2021)	Therapeutic	±	±
Activated charcoal + wood vinegar	Neonatal calves	6	Experimental	(Watarai et al., 2008)	Therapeutic	+	+
	Neonatal goat kids	40	Natural	(Paraud et al., 2011)	Prophylactic	±	±
Anti-IL-10 egg yolk antibody	Neonatal calves	133	Natural	(Raabis et al., 2018)	Prophylactic	–	–
Artificial sweetener/Glucagon-like peptide	Neonatal calves	24	Experimental	(Connor et al., 2017)	Prophylactic	±	±
Bobel-24 (anti-inflammatory drug)	Neonatal lambs	37	Experimental	(Castro-Hermida et al., 2008)	Prophylactic	±	±
					Therapeutic	±	–
Bovine/Ovine colostrum	Neonatal calves	12	Experimental	(Fayer et al., 1989)	Prophylactic	±	±
		30	Experimental	(Slacek et al., 1996)	Prophylactic	±	±
		12	Experimental	(Perryman et al., 1999)	Prophylactic	+	+
		10	Experimental	(Askari et al., 2016)	Prophylactic	+	+
		30	Natural	(Kacar et al., 2022)	Prophylactic	+	+
	Neonatal lambs	32	Experimental	(Naciri et al., 1994)	Prophylactic	+	±
	Gnotobiotic piglets	21	Experimental	(Tzipori et al., 1994)	Therapeutic	–	–
Bovine interleukin-12 (recombinant)	Neonatal calves	20	Experimental	(Pasquali et al., 2006)	Prophylactic	–	–
Bovine serum concentrate	Neonatal calves	24	Experimental	(Hunt et al., 2002)	Prophylactic	±	±
Bovine leukocyte extract	Neonatal calves	9	Experimental	(Fayer et al., 1987)	Prophylactic	–	–
Clinoptilolite	Neonatal lambs	30	Experimental	(Dinler Ay et al., 2021)	Prophylactic	+	+
					Therapeutic	+	+
Chitosan	Neonatal lambs	32	Experimental	(Aydogdu et al., 2019)	Therapeutic	±	±
Phytogenic extracts and essential oils	Neonatal calves	43	Experimental	(Olson et al., 1998)	Prophylactic	–	–
		41	Natural	(Weyl-Feinstein et al., 2014)	Prophylactic	+	±
		91	Natural	(Katsoulos et al., 2017)	Prophylactic	±	–
		30	Natural	(Volpato et al., 2019)	Prophylactic	–	–
		26	Experimental	(Mendonca et al., 2021)	Prophylactic	±	±
Probiotics (lactic acid producing bacteria)	Neonatal calves	134	Natural	(Harp et al., 1996)	Prophylactic	–	–
		30	Natural	(Fernandez et al., 2020)	Prophylactic	±	–
		44	Natural	(Stefańska et al., 2021)	Prophylactic	±	±
Yeast fermentation products	Neonatal calves	123	Natural	(Velez et al., 2019)	Prophylactic	–	–

“+” = complete cure; “-” = no demonstrable effect; “±” = partial cure or relapse after treatment discontinuation.

#### 3.2.1 Anticoccidials

##### 3.2.1.1 Halofuginone lactate

Halofuginone lactate, a prolyl-tRNA synthetase inhibitor, is a synthetic quinazolinone coccidiostat primarily used in veterinary medicine for the prevention and treatment of *Eimeria* infections in avian species. This medication is licensed for veterinary use in cattle against cryptosporidiosis in several European countries as well as Canada, although it is not labeled for use in the United States. Halofuginone lactate has a narrow safety index and is contraindicated in dehydrated animals suffering from diarrhea: clinical signs typical of cryptosporidiosis in neonatal animals. Hence, this drug is not suitable for therapeutic purposes and is generally used as a prophylactic to prevent cryptosporidial diarrhea in newborn farm animals. Recently, Brainard and others conducted a systematic review of literature and used meta-analysis to evaluate key outcomes such as oocyst shedding, diarrhea, mortality, and weight gain for the treatment of calf cryptosporidiosis with halofuginone lactate. The authors concluded that prophylactic halofuginone treatment was associated with significantly lower incidence of oocyst shedding, diarrhea burden, and mortality especially when the treatment was started early in life (Brainard et al., 2021). Furthermore, Giadinis et al. conducted two extensive field trials in Greece and found the drug to be effective in preventing and treating cryptosporidiosis, and reducing deaths associated with the disease in neonatal lambs and goat kids (Giadinis et al., 2007; Giadinis et al., 2008).

A number of early reports suggested that halofuginone showed effectiveness in protecting young ruminants from severe cryptosporidiosis, but relapses occurred after treatment discontinuation in calves (Villacorta et al., 1991; Naciri et al., 1993; Peeters et al., 1993; Lefay et al., 2001), lambs (Naciri and Yvore, 1989; Causapé et al., 1999), and goat kids (Chartier et al., 1999), questioning the effectiveness of the preventative treatment. Moreover, although halofuginone lactate treatment reduces oocyst shedding in infected animals, it fails to provide complete protection and cure, implying that treatment along with good animal husbandry practices including individual housing, proper hygiene measures, and suitable disinfection are required to prevent environmental contamination and disease transmission among animals on farms (Joachim et al., 2003; Jarvie et al., 2005; Klein, 2008; De Waele et al., 2010; Trotz-Williams et al., 2011; Keidel and Daugschies, 2013). Likewise, a few studies also showed some efficacy in reducing excretion of *Cryptosporidium* oocysts in treated animals as compared to untreated controls, but no significant effect on the prevalence of diarrhea or body weight gain was noted (Causapé et al., 1999; Lallemand et al., 2006; Trotz-Williams et al., 2011; Almawly et al., 2013). Interestingly, preventive treatment with halofuginone lactate was also found to be associated with reduced weight gain in calves (Niine et al., 2018; Velez et al., 2019). Thus, the preventive and therapeutic effectiveness of halofuginone lactate in animals remains controversial.

##### 3.2.1.2 Decoquinate

Decoquinate is a quinolone coccidiostat most used for controlling coccidiosis in ruminants and poultry. This drug inhibits the mitochondrial respiration by blocking electron transport in *Eimeria* parasites (Wang, 1976). Decoquinate produces limited-to-no clinical and parasitological response when used preventatively before the development of signs and symptoms of cryptosporidiosis in experimentally or naturally infected calves (Redman and Fox, 1993; Moore et al., 2003; Lallemand et al., 2006). However, it significantly reduces oocyst shedding and severity of cryptosporidiosis in neonatal kids, but without complete eradication of infection (Mancassola et al., 1997; Ferre et al., 2005).

##### 3.2.1.3 Lasalocid

Lasalocid is an ionophore antibiotic and a coccidiostat that is commonly used as a feed additive for promoting growth and preventing coccidiosis in ruminants. This drug has been used as a prophylactic or therapeutic to treat *Cryptosporidium* infections in calves. Based on anecdotal reports, short-term dosing (3-4 days) of lasalocid (6-15 mg/kg/day) was effective in treating severe cryptosporidiosis in calves (Gobel, 1987a; Gobel, 1987b; Sahal et al., 2005). However, mortality and serious side effects resulting from lasalocid toxicosis have been described in animals when long-term therapy or a dose higher than the label dose was used as a preventative for cryptosporidiosis (Moon et al., 1982; Benson et al., 1998). More recently however, Murakoshi and others demonstrated a highly beneficial effect of lasalocid, without any side effects, when used at a lower dose (3 mg/kg/day) to prevent calf cryptosporidiosis. But the treatment was not found to be protective after the 7-day dosing period (Murakoshi et al., 2014).

##### 3.2.1.4 Sulfonamides

Sulfonamides are broadly active antimicrobial agents that inhibit dihydropteroate synthase, an enzyme involved in folate synthesis (Henry, 1943). They have been widely used in veterinary medicine to prevent coccidiosis and treat bacterial infections in animals and poultry. However, prophylactic or therapeutic treatment of natural or experimental cryptosporidiosis with a variety of sulfonamides and potentiated sulfonamides including sulfadimidine, sulfadimethoxine, and cotrimoxazole (trimethoprim in combination with sulfamethoxazole) has failed miserably in calves (Moon et al., 1982; Fischer, 1983; Fayer, 1992; Joachim et al., 2003; Nasir et al., 2013) and goat kids (Naciri et al., 1984; Koudela and Bokova, 1997).

#### 3.2.2 Paromomycin

In addition to humans, paromomycin has also been extensively tested for anti-*Cryptosporidium* efficacy in various food animals. However, as has been the case in humans, results have been varied and the treatment failed to achieve complete parasitological cure in most studies. Prophylactic administration of paromomycin was found to decrease the duration and severity of diarrhea as well as the duration and intensity of oocyst shedding in calves experimentally infected with *C. parvum* (Fayer and Ellis, 1993). Similar positive results were reported in a controlled-blind field trial of natural infection in calves, but the treated group started shedding oocysts and developed diarrhea after the treatment withdrawal (Grinberg et al., 2002). However, paromomycin does seem to be more effective in small ruminants. Treatment has been shown to reduce both cryptosporidial oocyst output and severity of clinical signs, when used prophylactically in neonatal goat kids (Mancassola et al., 1995; Chartier et al., 1996; Johnson et al., 2000) and therapeutically in neonatal lambs (Viu et al., 2000). This agent was also proven to be therapeutically effective against moderate cryptosporidiosis but ineffective against severe cryptosporidiosis in infected gnotobiotic piglets (Tzipori et al., 1994; Theodos et al., 1998). However, paromomycin, like many aminoglycosides, is potentially nephrotoxic and detrimental effects on growth have been observed after treatment in young animals (Viu et al., 2000). In addition, the drug is expensive and therefore, its use in agricultural animals is impractical.

#### 3.2.3 Nitazoxanide

Nitazoxanide, the only licensed treatment available in humans, has also been tested for efficacy in animal cryptosporidiosis, although reports on treatment outcomes have been conflicting. While Ollivett and others found this medication to significantly reduce the duration of oocyst shedding and clinical severity in experimentally infected calves as compared to the placebo treated group (Ollivett et al., 2009), another controlled study found no prophylactic or therapeutic effect of nitazoxanide on clinical appearance or oocyst excretion in calves infected with *C. parvum* (Schnyder et al., 2009). Furthermore, while nitazoxanide reduced oocyst shedding in experimentally challenged newborn goat kids, no reduction in mortality rates or improvement in weight gains were recorded in the treated groups compared with the control group (Viel et al., 2007). Importantly, the authors of this study suggested that the mortalities seen in kid neonates in the nitazoxanide treated groups were caused by severe drug toxicity (Viel et al., 2007). In the gnotobiotic piglet diarrhea model, nitazoxanide demonstrated only partial efficacy at high doses in reducing *C. parvum* oocyst shedding, induced drug-related diarrhea, and was not as effective as paromomycin (Theodos et al., 1998). In another study performed in the same animal model but infected with *C. hominis*, nitazoxanide reduced diarrhea and oocyst shedding in only the initial phase of treatment and had no clinical or parasitological effect at the later stages of the disease (Lee et al., 2017).

#### 3.2.4 Macrolides

Macrolides have been evaluated as anti-*Cryptosporidium* agents in a range of animals. Azithromycin significantly suppressed *Cryptosporidium* oocyst shedding and resulted in significant clinical improvement and weight gain in naturally infected dairy calves when used as a therapeutic at high doses, but high costs of treatment are a concern (Elitok et al., 2005). Similar reports of azithromycin efficacy against *C. parvum* infection in calves (Nasir et al., 2013) and a buffalo calf (Maurya et al., 2016) have also been published. Treatment of gnotobiotic neonatal piglets infected with *C. hominis* alleviated clinical disease only for the first few days and azithromycin treated piglets exhibited no reduction of oocyst excretion compared with untreated animals (Lee et al., 2017). In combination with nitazoxanide, azithromycin led to significant clinical improvement in infected piglets but did not eliminate oocyst excretion after producing a transient initial reduction in oocyst shedding in treated animals (Lee et al., 2017).

Experience with other macrolides has also been mixed. While tilmicosin failed to prevent severe cryptosporidiosis in newborn kids raised on a commercial dairy goat farm (Paraud et al., 2010), tylosin was found to be therapeutically effective in reducing fecal oocyst excretion and clinical signs of disease in naturally infected calves (Duru et al., 2013).

#### 3.2.5 Other treatments

##### 3.2.5.1 Immunotherapy

Passive immunotherapy using bovine colostrum or bovine serum concentrate containing specific antibodies to *Cryptosporidium* provided only partial protection against cryptosporidiosis by reducing duration of diarrhea and oocyst shedding in experimentally infected calves (Fayer et al., 1989; Slacek et al., 1996; Hunt et al., 2002). However, much better protection from *Cryptosporidium* infection was noted in some studies after prophylactic oral administration of bovine/ovine colostrum comprising of anti-cryptosporidial antibodies in infected calves and lambs (Naciri et al., 1994; Perryman et al., 1999; Askari et al., 2016; Kacar et al., 2022). Moreover, therapeutic administration of hyperimmune colostrum-immunoglobulin in experimentally infected gnotobiotic piglets reduced oocyst shedding but had little-to-no effect on diarrhea and intestinal mucosal damage caused by the parasite (Tzipori et al., 1994). Similarly, preventive treatment of experimentally induced calf cryptosporidiosis by recombinant bovine interleukin-12 (rBoIL-12) or lymphocyte extracts from immunized calves failed to provide prophylaxis (Fayer et al., 1987; Pasquali et al., 2006).

##### 3.2.5.2 Adsorbents

Oral intestinal adsorbents have been used worldwide as a remedy to treat diarrhea of various causes. A product consisting of activated charcoal and wood vinegar was found to be highly effective in treating experimental *C. parvum* infection in calves (Watarai et al., 2008) and provided partial protection to newborn kids against natural infection (Paraud et al., 2011). More recently, activated charcoal also showed a partial curative effect on neonatal calf diarrhea caused mainly by *C. parvum* at a commercial calf-raising farm (Ross et al., 2021). Similarly, another adsorbent clinoptilolite also demonstrated a good prophylactic and therapeutic effect against *C. parvum* in experimentally infected lambs (Dinler Ay et al., 2021). These adsorbents seem to be effective against cryptosporidial infections probably because of their potential to adsorb and thereby trap parasites and prevent host cell invasion. Although this adsorption principle has been demonstrated in an *in vitro* adsorption test (Watarai et al., 2008), the same needs to be confirmed in further *in vivo* studies.

##### 3.2.5.3 Polysaccharides

Cyclodextrins are cyclic oligosaccharides that are commonly used as drug excipients to enhance the solubility, safety, stability, and bioavailability of drugs. After showing some unexpected activity against *C. parvum* experimental infection in mice, β-cyclodextrin has been tested for both prophylactic as well as therapeutic efficacy against cryptosporidiosis in young ruminants with results showing that the preventive effect is greater than the curative one. While β-cyclodextrin showed partial efficacy in reducing diarrhea and oocyst shedding in naturally infected calves (Castro-Hermida et al., 2001a), this drug reduced mortality and produced an even better clinical and parasitological response in infected lambs under field conditions (Castro-Hermida et al., 2001b). Another drug of this class, α-cyclodextrin was tested for prophylactic effectiveness in experimentally infected neonatal kids and showed some reduction in the intensity of infection and oocyst shedding, but almost half treated kids died probably due to drug-related side effects (Castro-Hermida et al., 2004).

Chitosan, a natural linear polysaccharide has also been investigated for efficacy in *C. parvum* infected lambs. Therapeutic treatment after the onset of disease improved clinical signs and fecal consistency, and reduced oocyst excretion, but did not eliminate cryptosporidiosis completely in treated lambs (Aydogdu et al., 2019).

Researchers have suggested various modes of action of polysaccharides such as cyclodextrins and chitosan in controlling viral, bacterial, and parasitic infections that involve use of their antimicrobial properties, osmotic properties, and cholesterol-sequestering ability, among others (Aydogdu et al., 2019; Braga, 2019). It is likely that, in the case of *Cryptosporidium*, these polysaccharides might form a protective film over the intestinal surface due to their adhesive properties, which may act as a physical barrier and prevent cell invasion by parasites. However, to date, the exact mechanism of action of these pharmaceutical agents against *Cryptosporidium* remains unknown.

##### 3.2.5.4 Natural plant-based products

Various natural products like phytogenic extracts, essential oils, and phytobiotics have been used to treat animal cryptosporidiosis, but with unconvincing results. A randomized controlled study evaluated allicin, a sulfur-containing component of garlic, in experimentally infected neonatal calves and found it to have no effect on the duration of diarrhea or weight gain in treated calves (Olson et al., 1998). Another study conducted in Israel showed that a concentrated pomegranate extract feed supplement partially reduced clinical signs and fecal oocyst counts in natural calf cryptosporidiosis (Weyl-Feinstein et al., 2014). Similarly, experimentally infected calves receiving plant-based isoquinoline alkaloids as feed additive suffered from less intense diarrhea for a shorter period but shed similar number of oocysts daily compared with the control group (Mendonca et al., 2021). Furthermore, administration of essential oils or essential oil-based phytogenic products to newborn calves also failed to produce any preventive effect on parasite shedding in infected calves (Katsoulos et al., 2017; Volpato et al., 2019).

##### 3.2.5.5 Probiotics

A few animal studies suggest some potential for the use of probiotics for prophylactic treatment of cryptosporidiosis, though bacterial mechanisms involved in protection against *Cryptosporidium* infection are not known. Daily oral administration of lactic acid producing bacteria for 10 consecutive days to *C*. *parvum* infected dairy calves had limited effect on clinical signs and no effect on parasite abundance (Harp et al., 1996; Fernandez et al., 2020), although a partial reduction in the severity of diarrhea, prevalence of cryptosporidial infection, and oocyst excretion was noted when probiotics combined with phytobiotics were dispensed to calves under field conditions (Stefańska et al., 2021). Likewise, feeding of yeast fermentation products had no clinical and parasitological benefits in bovine cryptosporidiosis (Velez et al., 2019).

##### 3.2.5.6 Miscellaneous treatments

Apart from drugs that have a direct anti-parasitic effect, other medications that have no known anti-*Cryptosporidium* activity but act by improving the symptoms of cryptosporidiosis have also been tested in animals. Such drugs might show some reduction of parasite load in young animals probably by relieving the symptoms of disease and allowing natural host immunity to develop and act against the infection. One such anti-inflammatory drug, Bobel-24, was unable to completely prevent or treat experimentally induced *C. parvum* infection in neonatal lambs but showed some prophylactic efficacy in reducing the duration and intensity of oocyst shedding and the presence of diarrhea (Castro-Hermida et al., 2008). Also, preventive administration of anti-IL-10 egg yolk antibodies for 11 days had no effect on the prevalence of *Cryptosporidium* infection in calves reared under field conditions (Raabis et al., 2018). In another study, administration of glucagon-like peptide 2 or artificial sweetener therapy before a low-level experimental *C. parvum* exposure reduced severity of diarrhea, fecal oocyst excretion, and intestinal pathology in neonatal calves (Connor et al., 2017).

## 4 New potential treatments for humans and animals

So far, no satisfactory prophylactics or therapeutics are available for the prevention or treatment of severe cryptosporidiosis in humans and animals. The limited progress made in this field can be directly attributed to the limited genetic tractability of *Cryptosporidium*, lack of conventional apicomplexan targets, as well as the unique intracellular but extracytoplasmic location within the host cells. Furthermore, the lack of reliable cell culture platforms and limited availability of technical tools to study the parasite in biological systems, lead to an inadequate knowledge about the host-parasite interactions (Checkley et al., 2015; Innes et al., 2020). Nevertheless, breakthrough genetic modification of the parasite that has been made recently has advanced *Cryptosporidium* research, although the approach is complicated compared to methods developed for other apicomplexan parasites, as it requires the passage of the transgenic parasites in laboratory animals (Vinayak et al., 2015). Importantly, some significant progress has been made in generating genetically modified *C*. *parvum* strains *in vitro*, using mouse-derived intestinal organoid cultures grown in a modified air-liquid-interface system, that enables the completion of the life cycle and produces viable oocysts that are infectious in cell culture and immunocompromised mice (Wilke et al., 2019; Wilke et al., 2020). Indeed, recent advances in genetic manipulation and culture of *Cryptosporidium* have resulted in substantial progress in anti-*Cryptosporidium* drug discovery in recent years, and several compounds of preclinical, lead, and late lead status have emerged from target-based and phenotypic screens and are currently in development (Love and Choy, 2021).

*C. parvum* infects both humans and cattle as natural hosts, with the human disease closely resembling the one found in neonatal calves (Santín and Trout, 2007). Thus, the use of the neonatal calf infection model is highly recommended for assessment of efficacy of candidate compounds before advancement to human clinical trials and should ensure the safety and efficacy of promising compounds in both humans and livestock. Recently some compounds have shown promising efficacy in treating cryptosporidiosis in natural animal host models including the neonatal calf model without any major safety issues. These include bumped kinase inhibitors (BKIs), pyrazolopyridine-based KDU731, triazolopyradizine MMV665917, benzoxaborole AN7973, and compound 2093 (**Table 4**).

BKIs inhibit the *Cryptosporidium parvum* calcium-dependent protein kinase 1 (CpCDPK1), an enzyme that is essential for host cell invasion and does not have any mammalian analogs (Van Voorhis et al., 2021). Recent studies have assessed novel BKIs as a possible cure for cryptosporidiosis. In one study, Lendner et al. evaluated a bumped kinase inhibitor BKI-1294 for efficacy against *C. parvum* in experimentally infected neonatal calves and concluded that BKI-1294 reduced oocyst shedding but had no effect on diarrhea and dehydration in treated calves (Lendner et al., 2015). In another study, Schaefer and others demonstrated that BKI-1294 significantly improved clinical appearance, diarrhea, and parasitological outcomes but failed to eliminate diarrhea and other clinical symptoms of bovine cryptosporidiosis (Schaefer et al., 2016). Nonetheless, another CpCDPK1 inhibitor, BKI-1369, has emerged as an encouraging lead compound for anti-*Cryptosporidium* therapy in animals (Van Voorhis et al., 2021). This compound has shown promising efficacy against cryptosporidiosis in both the *C. parvum* infected neonatal calf, and the *C. hominis* infected gnotobiotic piglet models (Hulverson et al., 2017; Lee et al., 2018). Unfortunately, these BKIs possess potent human *Ether*-*à*-*go*-*go*-Related *Gene* (hERG) inhibitory activity, which is associated with a potentially fatal disorder called long QT syndrome and cardiotoxicity in humans, effectively removing them from the anti-cryptosporidial drug development pipeline for humans (Van Voorhis et al., 2021). Nevertheless, BKI-1369 displayed both efficacy and safety in the neonatal calf model with a 30-fold reduction in total oocyst excretion and, therefore, calls for additional development as an anti-*Cryptosporidium* therapeutic for cattle (Hulverson et al., 2017).

The pyrazolopyridine derivative KDU731 is another promising anti-cryptosporidial drug candidate that inhibits the enzymatic activity of *Cryptosporidium* lipid kinase PI(4)K (phosphatidylinositol-4-OH-kinase) and is active against both *C. parvum* and *C. hominis*. Oral treatment with KDU731 resulted in significant reduction in oocyst shedding, duration of severe diarrhea, and dehydration without any adverse drug-related effects in neonatal calves experimentally infected with *C. parvum* (Manjunatha et al., 2017). Intriguingly, KDU731 displayed limited systemic exposure in pharmacokinetic analysis of the drug in *C. parvum*-infected calves, suggesting that systemic exposure may not be important for therapeutic efficacy.

Recently, a piperazine derivative MMV665917 with an unknown molecular mechanism of action (MMOA), was identified within the open access “Malaria Box” collection of antimalarial compounds and found to have potent *in vitro* activity against both *C. parvum* and *C. hominis* in addition to excellent *in vivo* anti-*Cryptosporidium* efficacy in mouse models of acute (IFN-γ KO) and chronic (NSG) cryptosporidiosis (Jumani et al., 2018). This compound was later tested in the neonatal calf model of cryptosporidiosis by Stebbins and colleagues and treatment resulted in rapid resolution and reduced duration of diarrhea, as well as a 94% reduction in total fecal excretion of cryptosporidial oocysts in treated calves compared with the control group (Stebbins et al., 2018). In another study conducted in the gnotobiotic piglet model, MMV665917 was shown to significantly reduce fecal *C. hominis* oocyst shedding, intestinal lesions, parasite colonization, and severity of diarrhea, compared with untreated control piglets (Lee et al., 2019). Unfortunately, like BKIs, this promising compound shows partial hERG inhibition and is potentially cardiotoxic in humans. However, similarities between the modes of action of BKIs and MMV665917 cannot be drawn based on this finding since hERG inhibition is generally an off-target effect and several compounds with diverse structures and modes of action are known to promiscuously block this channel (Witchel, 2011). Hence, studies to determine the MMOA of MMV665917 are needed to aid further lead optimization efforts to reduce the affinity for hERG binding.

Another compound that has been discovered by phenotypic screening of an antimalarial compound library for *Cryptosporidium* growth inhibitors is the 6-carboxamide benzoxaborole AN7973 (Lunde et al., 2019). Like MMV665917, AN7973 is active against both *C. parvum* and *C. hominis* in cell culture and shows promising efficacy in both the acute and chronic murine models of cryptosporidiosis but does not have a validated target in *Cryptosporidium*. In the calf clinical model of cryptosporidiosis, AN7973 demonstrated exceptional efficacy in reducing the total parasite fecal excretion by >90% with complete elimination of diarrhea and significant reduction in dehydration in treated calves. Furthermore, the compound possesses favorable safety, stability, and pharmacokinetic characteristics and does not inhibit hERG, a major liability for development of other potential anti-cryptosporidial therapeutics including BKIs and MMV665917 for the human disease (Lunde et al., 2019).

Aminoacyl-tRNA synthetase inhibitors have emerged as promising therapeutic candidates for targeting protein synthesis in *Cryptosporidium* for the development of anti-cryptosporidial drugs (Jain et al., 2017; Baragana et al., 2019; Buckner et al., 2019; Vinayak et al., 2020). Amongst these compounds, only the potent *Cryptosporidium parvum* methionyl-tRNA synthetase (CpMetRS) inhibitor, compound 2093, has been tested in the neonatal calf efficacy model of cryptosporidiosis so far (Hasan et al., 2021). In dairy calves experimentally infected with *C. parvum*, compound 2093 initially reduced total oocyst shedding, diarrhea, and dehydration during the first 4 days of infection but most treated calves relapsed later with a severe progressive disease indicating the likely emergence of drug resistance. Sequencing analysis of parasite DNA extracted from feces of relapsed animals revealed the presence of two mutant parasite strains with different single amino acid substitutions in the *CpMetRS* genomic locus that potentially conferred MetRS inhibitor resistance. Further genome editing, structural modeling, and enzymatic studies confirmed the spontaneous emergence of drug resistant *Cryptosporidium* parasites, an alarming finding that demands immediate attention (Hasan et al., 2021).

In addition to the above discussed compounds, several other promising compounds have been unveiled in the last few years and found to be effective in both *in vitro* and mouse models of cryptosporidial infection. These include but are not limited to benzoxaboroles (Swale et al., 2019; Bellini et al., 2020), 5-aminopyrazole-4-carboxamide-based BKIs (Huang et al., 2019), *C. parvum* prolyl-tRNA synthetase (CpPRS) inhibitors (Jain et al., 2017), *C. parvum* lysyl-tRNA synthetase (CpKRS) inhibitors (Baragana et al., 2019), *C. parvum* phenylalanyl-tRNA synthetase (CpPheRS) inhibitors (Vinayak et al., 2020), piperazine derivatives (Oboh et al., 2021), and glycolytic enzyme inhibitors (Li et al., 2019; Khan et al., 2022b). However, demonstration of efficacy and safety of these compounds in the neonatal calf and gnotobiotic piglet infection models is essential before further advancement to the next stages of development. Nevertheless, availability of multiple potential anti-cryptosporidial compounds is advantageous as a diverse pool of candidate compounds would be needed to account for the high attrition rate that is typical of drug development programs.

## 5 Vaccine development

Since efficacious anti-cryptosporidial drug options are currently lacking, vaccines could be a relevant option for the control of this disease. However, there are currently no vaccines available to prevent cryptosporidiosis. In any case, humans and animals with healthy immune systems suffer from a mild self-limiting illness and improve without treatment. Therefore, it is unclear whether vaccination is justified in these patient groups. However, vaccination could be particularly useful in preventing cryptosporidiosis in neonatal animals, immunocompromised individuals, and malnourished children living in underdeveloped countries. An effective vaccine should provide rapid long-lasting immunity in vaccinated individuals and minimize disease in livestock with a reduction in shedding of oocysts in feces thereby preventing the spread of the disease. A degree of cross-protective immunity against multiple species and subtypes, albeit less possible, will also be beneficial. The most viable strategy would be to vaccinate cattle, as they are the most significant contributors to contaminated manure globally (Vermeulen et al., 2017). However, it might be difficult to generate protective immunity in neonatal calves rapidly enough through active vaccination (Thomson et al., 2017). Therefore, passive immunization by transfer of anti-*Cryptosporidium* antibodies from immunized dams to calves through colostrum is a feasible alternate approach to protect them during the early days of life (Innes et al., 2020). Several immunogenic *Cryptosporidium* antigens, such as gp15, Cp15, and Cp23 that are involved in attachment or penetration of host cells, are being explored as vaccine candidates especially in the form of a multivalent vaccine, incorporating multiple antigens or antigenic epitopes (Mead, 2014; Innes et al., 2020). However, a major obstacle to the development of vaccines is our current limited understanding of the protective immune response against *Cryptosporidium* infection (Checkley et al., 2015).

## 6 Concluding remarks

The target patient population for anti-*Cryptosporidium* drug development is mainly comprised of young children, neonatal calves, and immunocompromised patients. These groups frequently suffer from co-morbidities due to their underdeveloped immunity or immunodeficiency and thus, there is an increased likelihood of such patients receiving other treatments. A highly safe pharmacological profile with a minimal risk of drug-drug interactions is, therefore, a key selection criterion for anti-*Cryptosporidium* drug candidates. Establishment of *in vitro* safety profiles of candidate compounds early in the drug development process is also crucial as it can help researchers filter out compounds with potential toxicity issues before they enter the costlier late stages of drug development. In addition to safety-related pharmacological properties, assessment of the absorption, distribution, metabolism, and excretion (ADME) properties of a lead compound is also critical to its initial selection and clinical success. Failure of translation of excellent *in vitro* efficacy into *in vivo* clinical potency may be caused by insufficient drug concentrations at the target site. Because cryptosporidiosis is primarily an enteric disease, optimal local gastrointestinal concentrations, in addition to systemic concentrations, might be essential for *in vivo* anti-*Cryptosporidium* efficacy of the compounds (Hulverson et al., 2017; Manjunatha et al., 2017; Stebbins et al., 2018; Lunde et al., 2019).

Modern drug-discovery projects utilize either a phenotype-based or target-based screening approach to identify lead candidate compounds for further development. *Cryptosporidium* drug discovery programs in the recent past have mostly used phenotypic screening methods to successfully discover or repurpose compounds with *in vitro* and *in vivo* activity against the parasite (Love et al., 2017; Jumani et al., 2018; Lunde et al., 2019; Li et al., 2020; Khan et al., 2022a). However, such an approach invariably results in the identification of candidate compounds that are difficult to optimize as their MMOA and structure-activity relationships (SAR) are generally unknown. This inflexibility typically leads to higher failure rates once a roadblock is reached in the drug development process. As such, molecular target identification and validation by various genetic and molecular “target deconvolution” methodologies are essential for hits identified from phenotype-based screens (Swinney and Anthony, 2011).

Another approach to anti-cryptosporidial drug discovery is to target biochemical pathways that are unique to the parasite and at the same time, essential for its survival, infection, or multiplication within the host. This strategy has also been used for anti-*Cryptosporidium* drug discovery, albeit less commonly than the phenotypic one. In the last few years, several enzymes have been identified as potential drug targets, including calcium-dependent protein kinases (Van Voorhis et al., 2021), aminoacyl-tRNA synthetases (Jain et al., 2017; Baragana et al., 2019; Buckner et al., 2019; Vinayak et al., 2020), lipid kinase PI(4)K (Manjunatha et al., 2017), and glycolytic enzymes (Witola et al., 2017; Eltahan et al., 2018; Zhang et al., 2018; Eltahan et al., 2019; Li et al., 2019; Velez et al., 2021; Khan et al., 2022b), among others. The advantage with this approach is that discovery of drug candidates with known MMOA and clearer SAR will create better opportunities for structure-based drug optimization. Indeed, while phenotypic screening has historically had more success in identifying first-in-class drugs, target-based screening has produced more best-in-class drugs (Swinney and Anthony, 2011). However, both approaches need to go hand in hand to identify safe and efficacious anti-*Cryptosporidium* lead compounds for further development.

Finally, the field will need to leverage the advantages of combination therapy for animal and human cryptosporidiosis to 1) increase the efficacy of treatment, 2) reduce the chances of host toxicity, and 3) prevent the inevitable rise of drug resistance in the future. As pointed out earlier in this review (**Table 3**), several drug combinations tested in the past have yielded superior results than monotherapy for treating *Cryptosporidium* infections in humans, though the number of studies evaluating drug combinations in animals is too small to draw a similar conclusion. Besides, given the success of combination therapies for other related apicomplexan diseases such as malaria, babesiosis, and toxoplasmosis, and the alarming acquisition of spontaneous drug resistant mutations during anti-cryptosporidial therapy by parasites in a recent study, more focus should be put on testing drug combinations against cryptosporidiosis in at-risk individuals.

## Author contributions

SK: conceptualization, literature review, and manuscript writing (original draft and revision). WW: conceptualization, supervision, manuscript writing (proof-reading, corrections, editing, and revision). All authors contributed to the article and approved the submitted version.
